# Traumatic cervical spinal cord injury in southeastern Norway: acute treatment, specialized rehabilitation referral and mortality

**DOI:** 10.3389/fneur.2024.1452194

**Published:** 2024-12-16

**Authors:** Tor Brommeland, Mona Strøm, Jalal Mirzamohammadi, Thomas Glott, Hege Linnerud, Pål Andre Rønning, Syed Ali Mujtaba Rizvi, Torjus Mogstad Holla, Birgitte Jensen Høydal, Donata Biernat, Mads Aarhus, Eirik Helseth

**Affiliations:** ^1^Department of Neurosurgery, Oslo University Hospital, Oslo, Norway; ^2^Spinal Unit, Sunnaas Rehabilitation Hospital, Bjørnemyr, Norway; ^3^Department of Neuroradiology, Oslo University Hospital, Oslo, Norway; ^4^Faculty of Medicine, Institute of Clinical Medicine, University of Oslo, Oslo, Norway

**Keywords:** spinal cord injury, cervical, neurotrauma, length of stay, surgery, specialized rehabilitation, mortality

## Abstract

**Background:**

Traumatic cervical spinal cord injury (cSCI) is a serious condition that requires a multidisciplinary treatment approach involving care at a neurotrauma center (NTC) and specialized rehabilitation. Contemporary population-based studies of cSCI are important for ensuring the quality and planning of health care approaches for these patients.

**Methods:**

This is a population-based cohort study of patients with traumatic cSCI who were admitted to the NTC in Southeast Norway between 2015 and 2022. The main outcome variables were length of stay (LOS), rate of surgical fixation/stabilization, rate of transfer to specialized rehabilitation, and 90-day mortality. Uni-and multivariate binary logistic regression analyses were used to investigate the effect of different covariates on LOS, transfer to specialized rehabilitation and 90-day mortality.

**Results:**

The median age of the 370 patients admitted to the NTC was 64 years, 75% were males, 40% had severe comorbidities, 45% had multiple injuries, and 67% underwent primary triage at a local hospital (LH). Surgical cervical stabilization/decompression was performed in 78% of the patients. The median LOS at the NTC was 9 days, and increasing LOS was significantly associated with young age, American Spinal Injury Association Impairment Scale (AIS) grade B, surgery and prolonged ventilatory support. Inpatient specialized rehabilitation was provided to 54% of patients. Receiving specialized rehabilitation was associated with younger age, preinjury independent living, more severe cSCI, no need for acute phase tracheostomy, and surgical stabilization/decompression. Only 6% of the octogenarians received specialized rehabilitation. The 90-day mortality rate was 13%, which was associated with older age, preinjury dependent living, more severe cSCI, upper cervical injuries, and days on ventilator and inversely correlated with LOS.

**Conclusion:**

Advanced age, especially among octogenarians, was significantly linked to a lack of specialized rehabilitation. Qualified physicians should assess all patients with cSCI for their need of rehabilitation and their potential to benefit from it. If the number of patients who are likely to respond to rehabilitation outnumbers the capacity of the rehabilitation center, we have two choices. Either guidelines for prioritization of patients for rehabilitation should be developed, or the capacity of the rehabilitation centers should be increased.

## 1 Introduction

The reported incidence of traumatic cervical spinal cord injury (cSCI) in Europe ranges from 0.5–2.6/100,000/year (1–5). Due to the relatively low incidence of cSCI, acute care is often centralized in a neurotrauma centre (NTC), and rehabilitation located to units specializing in spinal cord injuries (6, 7). Acute treatment is complex and requires the coordinated efforts of radiologists, anaesthesiologists, intensivists and surgeons and often requires variable lengths of hospital stay (LOS) in the NTC. The key elements of acute care in the NTC are monitoring and treatment in the intensive care unit (ICU) and surgical cervical stabilization/decompression. According to current guidelines, surgery is recommended within 24 h of injury (8, 9).

Rehabilitation is time-consuming and expensive, but there is a considerable amount of documentation showing that early rehabilitation in a specialized centre for SCI patients improves neurological outcomes, also for elderly patients (10–18) The advantages of direct transfer from an NTC to a specialized rehabilitation centre for patients with SCI are likely similar to those of direct transfer for patients with a traumatic brain injury (14, 19, 20). Recent studies have reported increasing numbers of traumatic SCIs among elderly people (2, 21–25). At present, in Norway, we have no information whether surgical stabilization/decompression and specialized rehabilitation are offered at the same rate to elderly patients with cSCI as to younger patients with cSCI.

Contemporary population-based prospective cohort studies involving traumatic cSCI patients are important for ensuring the quality and optimal planning of health care approaches for these patients. Here, we present a population-based (Southeastern Norway) cohort study of patients with traumatic cSCI covering the period from 2015–2022, focusing on the referral rate to NTC, rate of cervical stabilization/decompression, LOS at the NTC, rate of transfer to specialized rehabilitation and 90-day mortality. We hypothesize that the probabilities of both surgical intervention and specialized rehabilitation are age dependent.

## 2 Materials and methods

Oslo University Hospital (OUH) is the only NTC in Southeast Norway. Located in Oslo, this NTC serves all 20 local hospitals (LHs) that refer patients with head and cervical spine injuries. All trauma-related cervical procedures in this population were performed at OUH. The population within this region increased from 2.9 million in 2015 to 3.1 million in 2022. A detailed description of the Norwegian population concerning sex and age can be found at www.ssb.no (26). The patients included in this study were admitted to OUH for surgical or nonsurgical care for cSCI.

Acute management of patients with cSCI at OUH follows standard recommendations with ICU monitoring of vital functions, elevated mean arterial blood pressure (MAP) >85 mmHg for 5–7 days, ventilation support if needed, thrombosis prophylaxis with stockings and low-molecular weight heparin (LMWH), enteral nutrition if necessary, elimination surveillance and early rehabilitation (27–31). Acute surgery is recommended within 24 h of the injury (8). OUH has state-of-the-art operating theatres and is staffed 24/7 with qualified surgical and anaesthesiology teams. Standard intraoperative fluoroscopy was used for all procedures. A spinal navigation system (BrainLab) based on preoperative Computed Tomography (CT) images was utilized for cervical and upper thoracic pedicle screw placement. Patients with persistent neurological disability are routinely referred to a specialized rehabilitation centre for SCIs (Sunnaas Rehabilitation Hospital is the only specialized SCI rehabilitation unit in Southeast Norway).

All consecutive patients with traumatic cervical spine injury were prospectively registered in a quality control database. This database includes patients who were diagnosed with traumatic cervical spine injuries (C0/C1 to C7/Th1) via cervical CT and/or Magnetic Resonance Imaging (MRI) in Southeast Norway (32). All cervical fractures, discoligamentous injuries in need of stabilization (cervical collar or surgery), and all traumatic cSCIs were included. Only patients with an 11-digit unique Norwegian Social Security Number living within Southeast Norway were included.

From Jan 1, 2015, to December 31, 2022, 3,622 patients with cervical spine injury were included. Concomitant cSCI was observed in 387/3622 patients (10.7%), of whom 370 patients were admitted to OUH (the NTC) for acute care. Seventeen patients not transferred to the OUH had sensory myelopathy or minimal paresis (9 patients), severe comorbidities (4 patients), and nonsurvivable C0-C2 injury (2 patients) or were admitted to the LH after acute treatment of the cSCI abroad (2 patients). The study cohort thus comprised 370 consecutive patients who were admitted to OUH (the NTC) for acute management. Sixteen of these patients underwent acute cervical surgery before admission to OUH, 9 at hospitals abroad and 7 at NTCs located in other Norwegian regions.

The following data were retrieved from the database: date and time of injury, date of admission to OUH, primary hospitalization after injury (NTC or LH), sex, age at time of injury, preinjury American Society of Anaesthesiologists Physical Status (ASA) score (1: normal healthy; 2: mild systemic disease; 3: severe systemic disease; 4: life-threatening systemic disease), preinjury living status (independent or dependent), level of cSCI (C0-C3, C3-C5 or C5-Th1), cSCI classified according to the American Spinal Injury Association (ASIA) Impairment Scale (AIS) (33) into Grade A (complete)-B-C-D (incomplete), type of cSCI (central cord syndrome (CCS) or non-CCS), multiple injury (yes or no), date and time of surgical decompression/fixation, surgical approach (anterior, posterior, 360°), LOS at NTC (days), length of ICU stay (LICUS in days), length of ventilator treatment (LVT in days), tracheostomy (yes or no), discharge destination (home, LH, inpatient specialized rehabilitation hospital (Sunnaas Rehabilitation Hospital), nursing home), in-hospital death (yes or no), in-patient rehabilitation (yes or no), in-patient rehabilitation (direct transfer from NTC or indirect transfer via LH), and last follow-up date/date of death.

CCS was defined as a cSCI resulting in more pronounced paresis in the arms than in the legs (34). If a patient was on a ventilator for surgical treatment only and extubated immediately after surgery, the LVT was zero days.

Multiple injuries were recorded when traumatic brain injury [mild, moderate, or severe was diagnosed according to the head injury severity scale (HISS)] and/or an injury was confirmed on X-ray, CT, or ultrasound in one or more of the following regions: the face, thoracolumbar spine, chest, abdomen, pelvis or extremities. Skin injuries were not registered.

The study was approved by the OUH Data Protection Office (DPO approval no 23/28298). The need to obtain informed consent from patients was waived. The quality control database for traumatic cervical spine injuries in southeastern Norway is approved by the OUH Data Protection Office (DPO approval no 2014/12304).

### 2.1 Statistics

The data were summarized using frequencies for categorical data and median values for continuous data. The Wilcoxon rank-sum test, Kruskal–Wallis test and chi-squared test were used to compare continuous and categorical variables. In analysing the LOS as a dependent variable, we compared linear-, poisson-, gamma and negative binomial regression models to assess the model performance and assumptions. Negative binomial regression was selected because it provided the best performance in terms of Akaike Information Criterion (AIC) and addressed the key assumptions related to the LOS data. LOS is inherently a count variable, with an overdispersion characteristic (the variance exceeds the mean), which violates the assumptions of Poisson regression. The negative binomial model accommodates overdispersion by introducing a dispersion parameter, making it a better fit for the data. For binary outcomes such as discharge destination and 90-day mortality, uni-and multivariate binary logistic regression analyses were performed. Logistic regression is widely used for binary outcomes as it models the probability of an event occurring while adjusting for covariates. When analysing factors potentially associated with inpatient specialized rehabilitation, we excluded those who died within 30 days of injury. Kaplan–Meier plots were used to explore survival after cSCI. *p* values <0.05 were considered significant.

In the Forest plots the dot represents the point estimate of the effect size. The horizontal line around the dot represents the confidence interval (CI) of the effect size. A finding is statistically significant if the entire confidence interval does not cross the vertical dashed line at 0 (“the null hypothesis”). In the Forest plots, statistically significant findings are marked with red dots/lines.

## 3 Results

**Figure 1:**
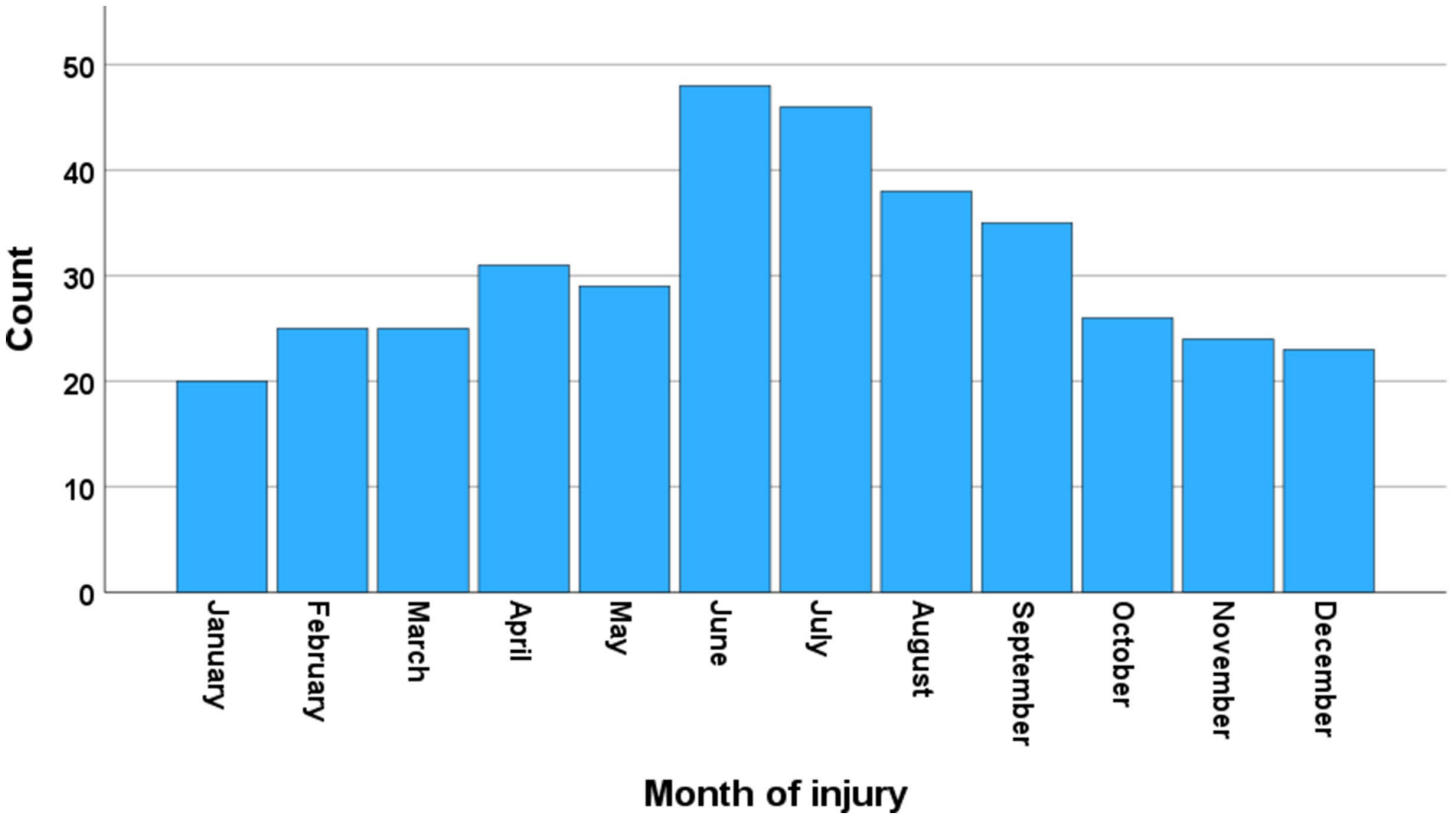
Seasonal variation. Number of patients with cervical spinal cord injury (cSCI) who were admitted to the neurotrauma center (NTC) during 2015–2022 according to the month of injury (*N* = 370).

From 2015–2022, 387 patients with cSCI were registered in our database. The study cohort included 370/387 (96%) consecutive patients with cSCI who were admitted to the NTC for acute management. The median age of the participants was 64 years (Inter Quartile Range (IQR) 48–74), 50% were ≥ 65 years of age (WHO definition of elderly), 75% were males, 40% had severe comorbidities (preinjury-ASA ≥3), 9% were not living independently, and 45% had multiple injuries. Direct transfer from the scene of the accident to the NTC was registered for 33% (123/370) of the patients, while 67% (247/370) underwent primary triage at a LH. The mean yearly number of patients was 46 (range 39–52). There was clear seasonal variation, with a peak during the summer months in Norway (Figure 1). The severity of cSCI according to the AIS was grade A in 17% of patients, grade B in 13% of patients, grade C in 24% of patients, and grade D in 46% of patients. The cSCI had a CCS phenotype in 158/370 (43%) of the patients, 34 of whom had AIS grade C and 124 of whom had AIS grade D. Additional patient characteristics are provided in (Table 1).

**Table 1:** Characteristics of patients with cervical spinal cord injury (cSCI) who were admitted to the regional neurotrauma center (NTC) between 2015 and 2022 (*N* = 370).

Characteristics	*N* (%)
Sex	
Male	277 (74.9)
Age	
≥ 65 years	184 (49.7)
Preinjury severe comorbidities	
ASA score ≥ 3	148 (40)
Preinjury living status	
Independent	329 (88.9)
Dependent	34 (9.2)
Unknown	7 (1.9)
AIS grade	
A – Complete	64 (17.3)
B – Sensory incomplete	48 (13)
C – Motor incomplete (muscle strength ≤ grade 3)*	89 (24.1)
D – Motor incomplete (muscle strength >grade 3)*	169 (45.7)
Max level of cSCI	
C0 – C3 above roots to phrenic nerve	48 (13)
C3 – C5 level roots to phrenic nerve	146 (39.5)
C5 – Th1 below roots to phrenic nerve	176 (47.5)
Multiple trauma	
Yes	166 (44.9)
Primary triage	
LH	247 (67)
NTC	123 (33)
Admitted ICU at NTC	
Yes	323 (87.3)
Ventilator therapy at NTC	
Yes	122 (33)
Tracheostomy at NTC	
Yes	49 (13.2)
Surgical fixation/decompression	
Yes (incl. 16 pts. operated other hospital)	288 (77.8)
Discharge destination	
Home	20 (5.4)
LH	171 (46.2)
Specialized rehabilitation (direct)	139 (37.6)
Nursing home	9 (2.4)
In-hospital death	31 (8.4)
Specialized inpatient rehabilitation	
Direct transfer from NTC (unbroken chain)	139 (37.6)
Indirect transfer via LH (broken chain)	61 (16.5)
Mortality	
Inpatient	31 (8.4)
90-day mortality	48 (13)

ASA score: American society of anaesthesiologists physical status classification system score; AIS: American Spinal Injury Association Impairment Scale; ICU: intensive care unit; LH: local hospital; NTC: neurotrauma center. *Medical Research Council (MRC) Scale for Muscle Strength. Grade 5: Normal. Grade 4: Movement against gravity and resistance. Grade 3: Movement against gravity. Grade 2: Movement of the limb but not against gravity. Grade 1: Visible contraction without movement of the limb. Grade 0: No visible contraction.

Fewer patients who were age ≥ 80 years were transported directly to the NTC after the injury (chi-square test, *p* = 0.004) (Table 2). The type of SCI and AIS grade did not vary significantly between age groups.

**Table 2:** Patients with cervical spinal cord injury (cSCI) who were admitted to the regional neurotrauma center (NTC) (*N* = 370).

Admission	< 65 years, *n* (%) *N* = 189	65–69 years, *n* (%) *N* = 51	70–79 years, *n* (%) *N* = 83	> 80 years, *n* (%) *N* = 47
Admitted to NTC				
Directly	70 (37)	16 (30)	30 (36)	7 (15)^*^
Surgery				
Yes	149 (79)	40 (78)	66 (79)	33 (70)
ICU stay				
Yes	165 (87)	45 (88)	76 (92)	37 (79)
Specialized rehabilitation				
Yes	133 (70)	29 (56)	38 (46)	5 (11)^*^
Mortality				
In-hospital	10 (5)	4 (8)	12 (15)	5 (11)
90-day	10 (5)	5 (9)	18 (21)	15 (32)^*^

Key variables stratified according to age group. NTC: neurotrauma center; ICU: intensive care unit. *Octogenarians significantly different from the other age groups.

### 3.1 Surgical procedures

Surgical decompression with or without stabilization was performed in 288/370 (78%) patients (Table 1), 16 of whom underwent acute surgery before admission to OUH. The rate of surgical procedures was the same across all age groups (Table 2). Anterior cervical stabilization was performed in 130/288 patients (45%), posterior stabilization in 75/288 patients (26%), combined anterior/posterior stabilization in 59/288 patients (21%), and posterior laminectomy only in 24/288 patients (8%). Among the 272 patients who underwent surgery at OUH, early surgery (<24 h) was performed in 41% (111/272) of patients. The median times from injury to the start of the operation according to AIS grades A, B, C and D were 18 h, 20 h, 27 h and 62 h, respectively.

Surgery was not performed in 82/370 (22%) patients with cSCI. The reasons for nonsurgical management were minor neurological deficits and/or no need for surgical decompression/stabilization in 39 patients, nonsurvivable injury in 26 patients, comorbidities resulting in the risk of complications superseding the potential benefit of surgery in 8 patients, root avulsion/brachial plexus injury as the main injury in one patient, and a knife stab lesion of the spinal cord in one patient. In the remaining 7 patients, the reason for choosing a nonoperative treatment strategy could not be identified.

### 3.2 Length of hospital stay at the NTC

The median LOS at the NTC was 9 days (IQR 6–12). ICU stay was documented for 323 out of 370 patients (87%), and the median length of ICU stay was 7 days (IQR 5–10). One hundred twenty-two of the 370 patients (33%) received ventilator support following surgery. The median LVT was 5 days (IQR 2–12). A tracheostomy was performed in 13% (49/370) of the patients.

**Figure 2:**
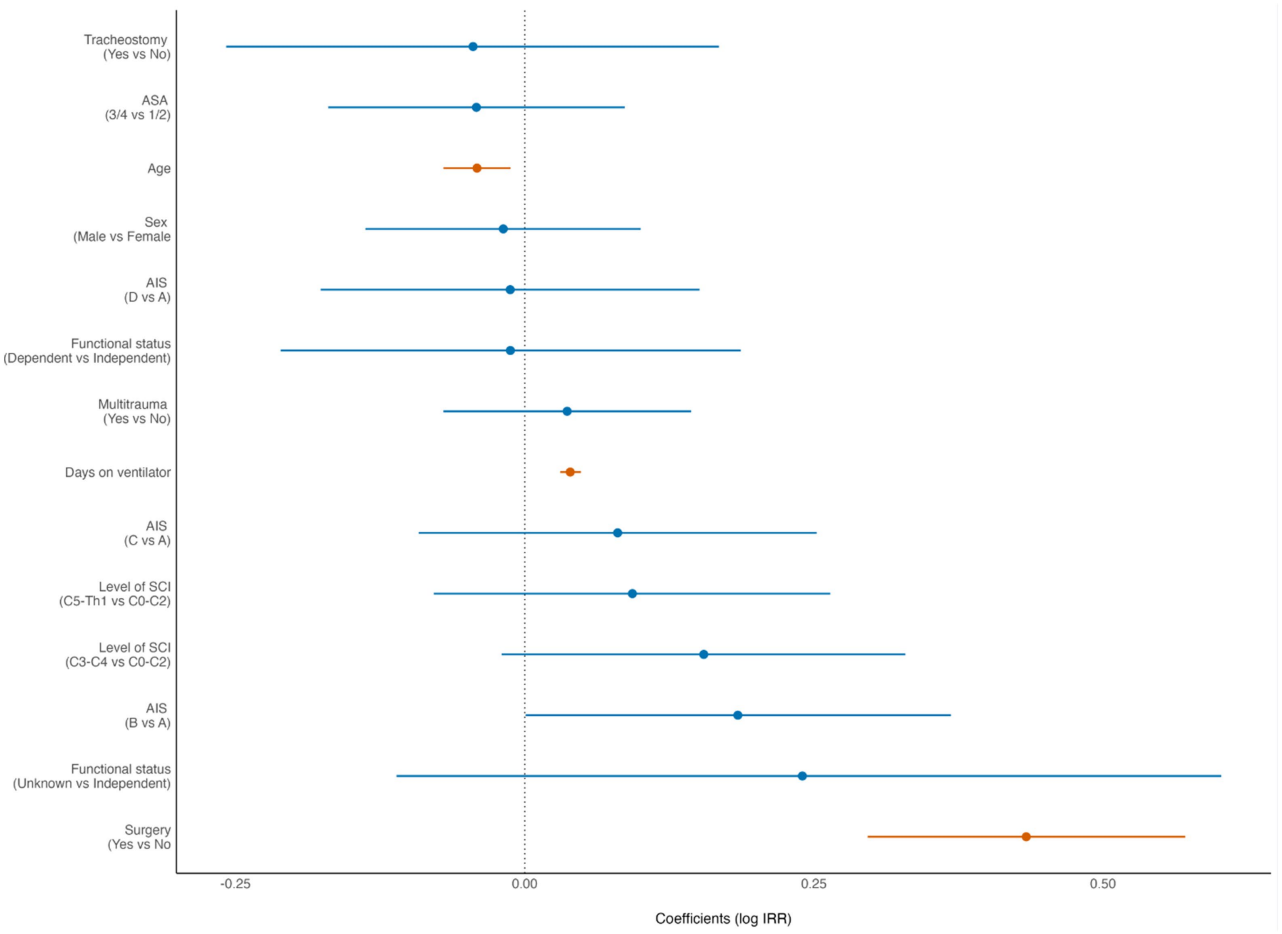
Forest plot from multivariate analysis of factors potentially associated with length of hospital stay (LOS) of patients with cervical spinal cord injury (cSCI) admitted to the neurotrauma center (NTC) during 2015–2022 (*N* = 370). ASA, American Society of Anaesthesiologists Physical Status score. AIS, American Spinal Injury Association Impairment Scale. IRR incidence rate ratio (negative binominal regression). Statistically significant findings are marked with red dots/lines.

According to the multivariable negative binomial regression analysis, increasing LOS was significantly associated with young age (*p* = 0.005), AIS grade B (*p* = 0.05), surgery (*p* < 0.001) and longer LVT (*p* < 0.001) (Figure 2).

### 3.3 Referral to specialized inpatient SCI rehabilitation

The primary discharge destinations were a LH for 46.2% of patients (171/370), a specialized inpatient SCI rehabilitation centre for 37.6% of patients (139/370), home for 5.4% of patients (20/370), and a nursing home for 2.4% of patients (9/370). Thirty-one of 370 (8.4%) patients died in the NTC (in-hospital death). Of the 171 patients who were transferred from the NTC to the LH, 61 patients were later referred for specialized inpatient SCI rehabilitation. Thus, a total of 200/370 (54%) of all patients with cSCI underwent specialized inpatient SCI rehabilitation. None of the 82 patients who were ≥ 80 years of age were directly transferred from the NTC to a specialized rehabilitation centre, and only 5/82 (6%) of the octogenarians were later transferred to a specialized rehabilitation centre.

**Figure 3:**
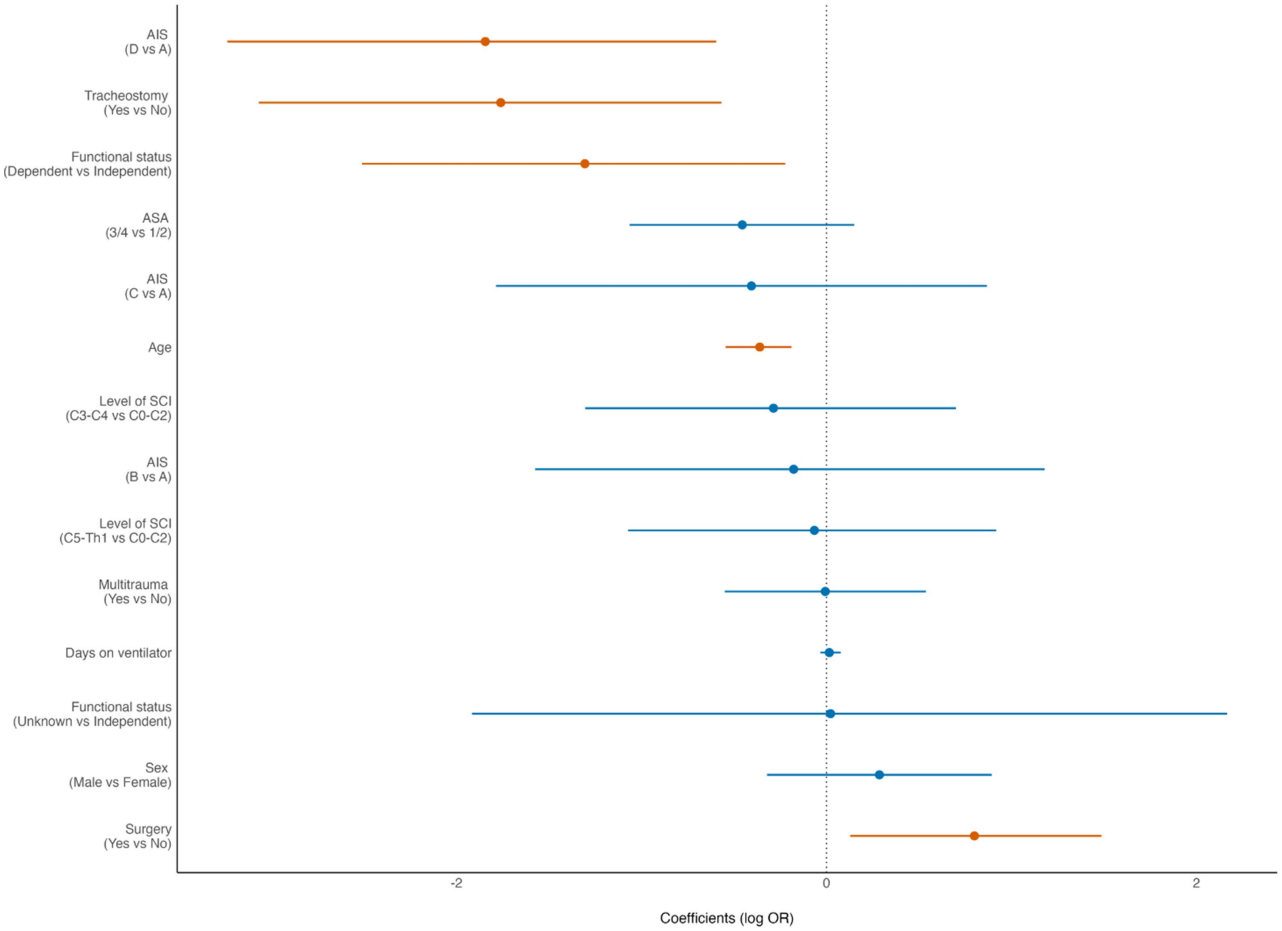
Forest plot from multivariate analysis of factors potentially associated with inpatient specialized rehabilitation in patients with cervical spinal cord injury (cSCI) admitted to the neurotrauma center (NTC) during 2015–2022 and who survived the acute phase (*N* = 329). ASA, American Society of Anaesthesiologists Physical Status score; AIS, American Spinal Injury Association Impairment Scale. OR, odds ratio (logistic regression). Statistically significant findings are marked with red dots/lines.

Of the patients who survived the acute phase (>30 days), 200/329 (61%) received inpatient specialized rehabilitation. For these patients, receiving specialized rehabilitation treatment was associated with younger age (*p* < 0.001), preinjury independent living (*p* = 0.024), severe cSCI (*p* = 0.006), no need for acute phase tracheostomy (*p* = 0.005), and surgery (*p* = 0.02) (Figure 3).

### 3.4 90-day mortality

**Figure 4:**
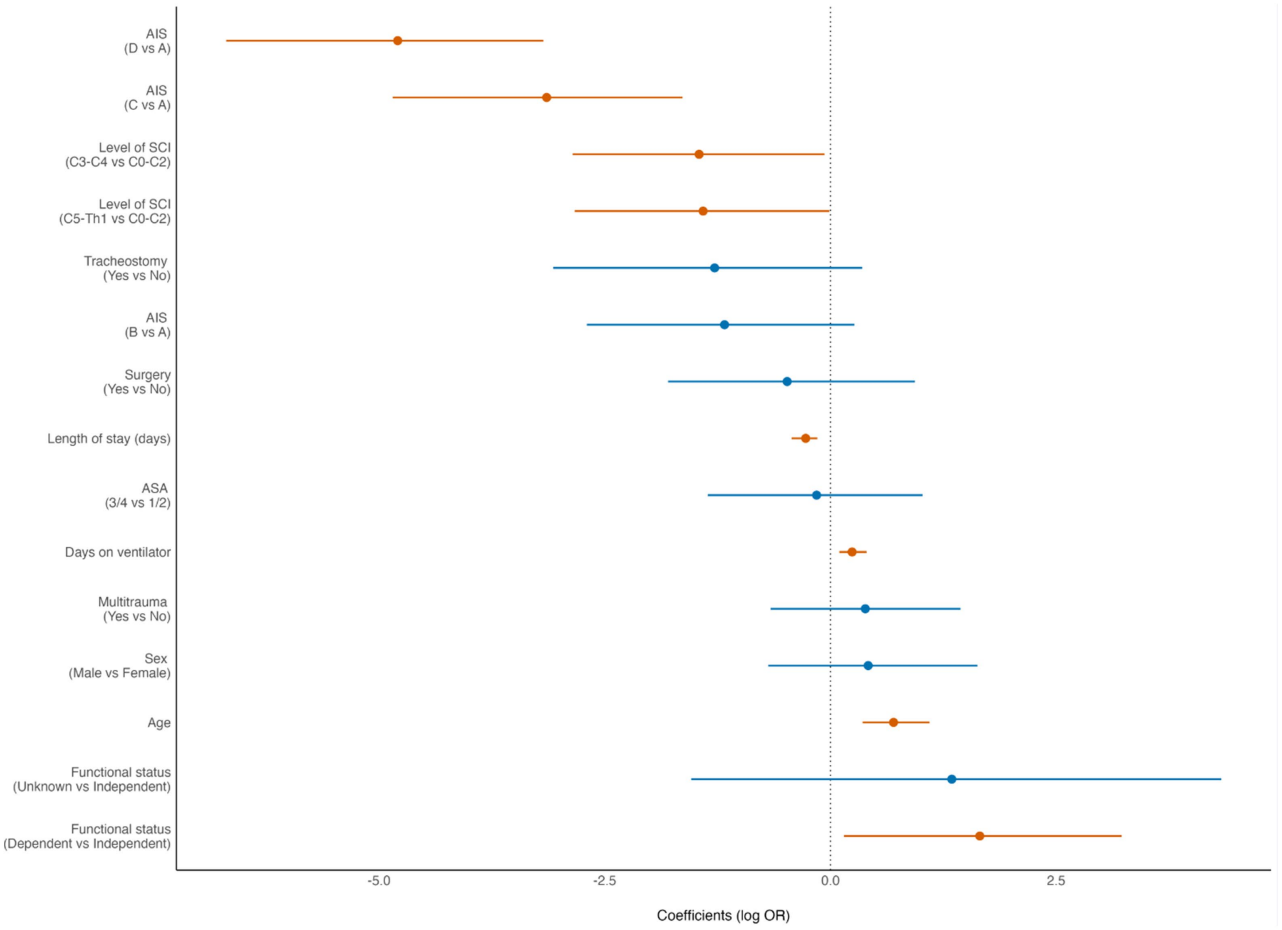
Forest plot from multivariate analysis of factors potentially associated with 90-day mortality of patients with cervical spinal cord injury (cSCI) admitted to the neurotrauma center (NTC) during 2015–2022 (*N* = 370). ASA, American Society of Anaesthesiologists Physical Status score; AIS, American Spinal Injury Association Impairment Scale; LOS, length of hospital stay. OR, odds ratio (logistic regression). Statistically significant findings are marked with red dots/lines.

**Figure 5:**
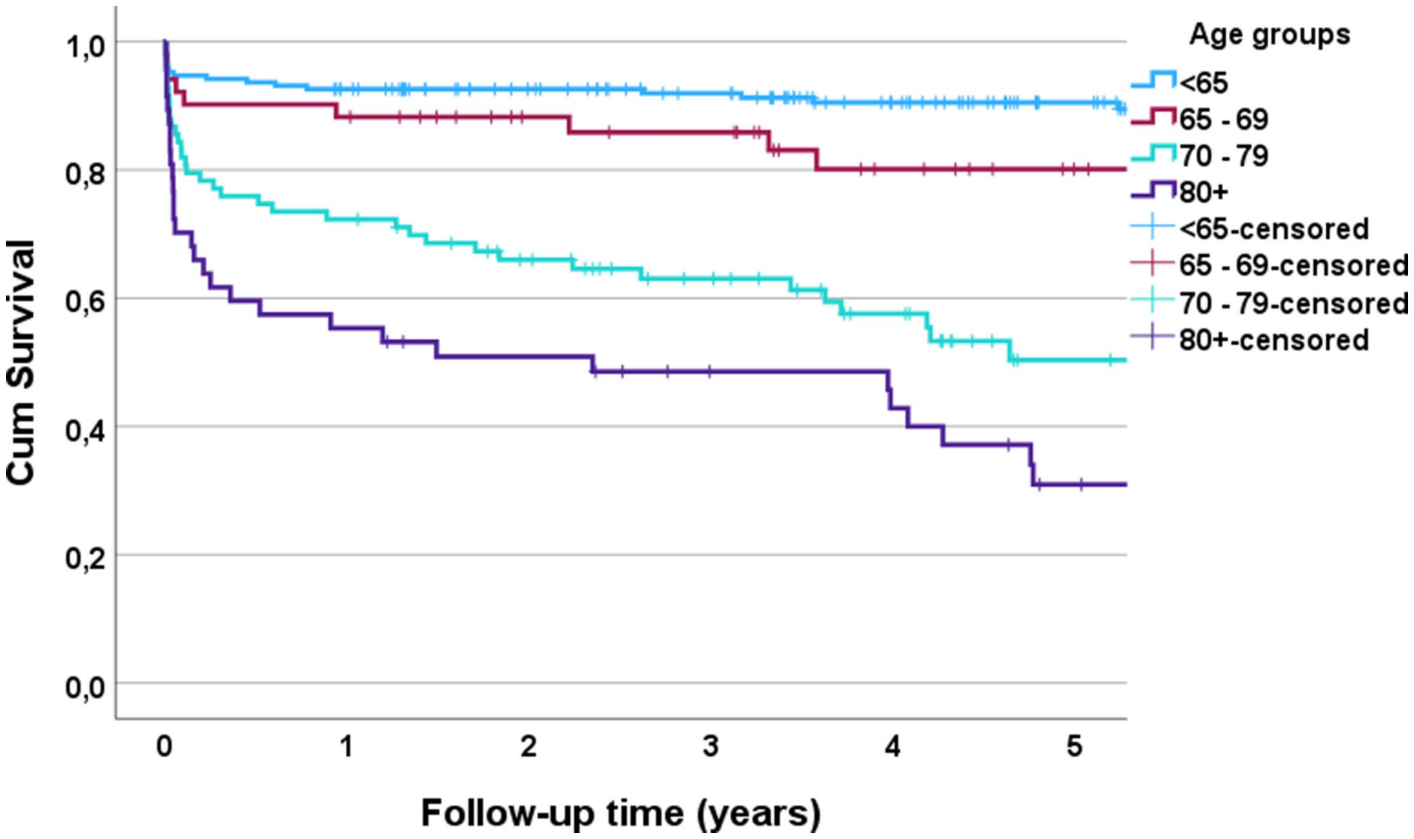
Kaplan Meier plot of survival after cervical spinal cord injury (cSCI) according to age for patients with cSCI admitted to the neurotrauma center (NTC) during 2015–2022 (*N* = 370).

The 90-day mortality rate for the entire cohort was 13% (48/370). According to multivariate binary logistic regression analyses, the mortality rate was associated with advanced age (*p* < 0.001), preinjury dependent living (*p* = 0.033), high-grade AIS (*p* < 0.001), C0–C3 injury (*p* = 0.039), LVT (*p* = 0.002) and was inversely correlated with the LOS (*p* < 0.001) (Figure 4). For patients who were > 80 years of age, the 90-day mortality rate was 32% (15/47). The Kaplan–Meier plot in (Figure 5) displays the effect of age on survival in patients with cSCI.

## 4 Discussion

Over the 8-year study period, 387 patients were diagnosed with cSCI in Southeast Norway. Among these patients, 96% were admitted to the regional NTC and composed the study cohort. The median age of the patients was 64 years, 83% had incomplete cSCI, 67% were referred after triage at a LH, the median LOS at the NTC was 9 days, 87% were treated in the ICU, and 78% underwent surgical decompression/fixation. The overall 90-day mortality rate was 13%, but it was 32% in octogenarians. Of the patients who survived the acute phase (>30 days), 61% received inpatient specialized rehabilitation. Advanced age, comorbidities and less severe cSCI were associated with reduced access to specialized rehabilitation. Only 6% of the octogenarians received specialized rehabilitation. This reflects a priority not to offer octogenarians specialized rehabilitation due to anticipated low rehabilitation potential. Whether capacity should be increased to include even the oldest age groups warrants further studies.

The number of patients with cSCI who were registered in Southeast Norway during the study period gives an estimated incidence of cSCI of 1.6/100,000/year, which is in line with the incidence of traumatic cSCIs reported in Europe (1–5). In our cohort, 50% of the patients were ≥ 65 years old. This finding was expected since several recent studies have reported increasing numbers of traumatic SCIs among elderly people (2, 21–25). This increase is likely attributable to the substantial increase in the number of elderly individuals overall (26) and the increased vulnerability of elderly people to sustain SCIs with relatively minor trauma compared to younger people (35). In particular, the occurrence of injuries resulting from falls has increased concurrently with the ageing of the overall population (36, 37).

### 4.1 Length of stay and surgical decompression/stabilization

Optimal treatment for SCI involves early clinical and radiological identification of the injury and transfer to an institution capable of multidisciplinary acute treatment (14, 19, 37). In Southeast Norway, 96% of patients were transferred to the NTC for acute treatment, indicating a need for NTC referral and a willingness for the evaluation/treatment of these patients at the NTC.

Monitoring vital functions such as respiration and blood pressure is essential in patients with cSCI (38). Patients with SCI should maintain a MAP between 75–80 and 90–95 mmHg for 5–7 days, often requiring an ICU stay for this purpose only (39, 40). Impaired respiration is frequent, either due to the cSCI itself or concomitant thoracic injuries such as pneumothorax, flail chest, or lung contusions. Ventilation difficulties secondary to aspiration pneumonia or ventilator-associated pneumonia are also common (41). Thus, observation in an ICU is mandatory for most of these patients. The median LOS at the NTC was 9 days, and a longer stay was significantly associated with younger age, cervical surgery, severe cSCI and LVT. This is in contrast to other studies demonstrating considerably longer stays at an NTC (42, 43). The shorter LOS for the elderly population was somewhat surprising because of the greater number of comorbidities in this patient group. A similar trend was found for the length of ICU stay and is also reported by others (43, 44). This may reflect limited ICU capacity and a less ambitious treatment approach for the elderly population.

Surgical cervical decompression/stabilization was performed in 78% of the patients, and surgery was performed at equal rates regardless of age. According to recently updated guidelines, surgery is recommended within 24 h of the injury (8). Early surgical intervention seem to affect the neurological outcome as well as LOS and hospitalization costs (45). The rate of surgery for patients with cSCI is much higher than that for patients with cervical spine fractures without SCI (32). The most common surgical procedure was anterior decompression and stabilization. Patients with AIS grades A and B were prioritized for surgery earlier than those with AIS grades C and D. This is probably partially due to patients with high AIS grades more often having severely unstable injuries and in need of urgent surgery. However, among the 272 patients who underwent surgery at OUH, early surgery (<24 h) was performed in only 47%. In a recently published study from our institution, the causes for delayed surgery was transfer to LH first, less severe AIS, high age and non-translational injury (46). Age has been demonstrated to affect timing of surgery also by others (47).

### 4.2 Specialized spinal cord injury rehabilitation

Direct transfer from the NTC to a specialized rehabilitation center is preferred, but a temporary stay at a LH may be necessary due to continued acute medical treatment or insufficient capacity at the rehabilitation hospital. Early rehabilitation at a specialized center seem to improve the final neurological outcome of SCI patients (14–16).

In our cohort of patients with cSCI, 54% received inpatient specialized rehabilitation, of whom 70% were transferred directly from the NTC to a rehabilitation center (unbroken chain) and 30% via the LH (broken chain). Of the patients who survived the acute phase (>30 days), 200/329 (61%) received inpatient specialized rehabilitation. Getting specialized rehabilitation was associated with younger age, preinjury independent living, more severe cSCI, no need for acute phase tracheostomy, and surgery. The decreased rate of referral to rehabilitation centers in elderly patients is also demonstrated by others (48). If this referral rate is related to a reduced need for or too low of a rehabilitation potential, it is acceptable and will prevent overtreatment and unnecessary use of limited health care resources. On the other hand, if it is associated with an insufficient capacity or a general belief among acute care physicians that elderly people do not respond to rehabilitation, changes may be warranted. Elderly patients who were able to live independently before injury and had few comorbidities seem to benefit from specialized rehabilitation after SCI in several studies (10–13, 49).

No patient aged ≥80 years was considered a candidate for direct transfer to a specialized rehabilitation center in our NTC and only 68% of these patients survived more than 90 days. Whether this represents the true course of cSCI in the oldest individuals of our population or is the result of a self-fulfilling prophecy is uncertain. Assuming that all octogenarians have a low rehabilitation potential and high mortality rate will possibly deprive some of these patients optimal treatment and should be the topic of future investigations with an adapted geriatric SCI program.

The assessment of rehabilitation potential in elderly patients with cSCI is complicated, and several factors need to be considered (44). Neurologically impaired muscle function is an important prognostic factor in patients with the potential to regain independence in activities through training (10). Patients need to have the cognitive ability to learn new skills and methods for performing activities of daily living. Thus, dementia and delirium are complications that reduce the potential of rehabilitation. Cardiopulmonary conditions are common and may limit the patient’s ability to perform even light physical activities. Comorbidities such as cancer, concomitant neurological diseases and generalized arthrosis may be of similar importance in elderly people. In patients with limited potential for rehabilitation, the aim is to compensate with assistive devices and prevent complications associated with the skin (pressure sores), urinary tract (infections and incontinence), bowel system (constipation and incontinence) and respiratory system (pneumonia, respiratory failure).

In our opinion, qualified physicians should assess all patients with cSCI for their need of rehabilitation and their potential to benefit from it. If the number of patients who are likely to respond to rehabilitation outnumbers the capacity of the rehabilitation center, we have two choices. Either guidelines for prioritization of patients for rehabilitation should be developed, or the capacity of the rehabilitation centers should be increased.

### 4.3 Mortality

The in-hospital and 90-day mortality rates were 8.4 and 13%, respectively. A recent meta-analysis of 21 studies by Sadeghi-Naini et al. (50) revealed an overall in-hospital mortality rate of 18% for subaxial cSCI. The large difference with respect to in-hospital mortality between our Norwegian study and “The World meta-analysis” is most likely explained by differences in patient populations and health care resources. Our results showed a significant association between 90-day mortality and advanced age, preinjury dependent living, cSCI severity, C0–C3 injury, and number of ventilator days. This finding is in line with other studies (51, 52). The risk of impaired respiration is greater for patients with C0–C3 cSCI injuries than for those with injuries below this segment (53). Interestingly, preinjury living status was associated with survival, while preinjury ASA score (comorbidity measure) was not. It is likely that the frailty score would be better than the ASA score. The frailty score reflects the patient’s comorbidities, functioning and physiological reserve capacity (44). Multiple traumas, which were registered for 45% of the patients, were not associated with increased mortality. The most likely explanation for this is that in this patient cohort, the most serious and defining injury was cSCI.

Of patients who were aged ≥80 years, 32% died within 90 days after their accident, thereby emphasizing the severity of this injury in octogenarians. In a large cohort of 1,340 elderly Canadian patients with SCI at any level, the expected in-hospital death rate was 16% for the entire cohort and as high as 86% for those aged ≥80 years with conservatively managed cervical AIS grade A (54).

### 4.4 Strengths

This study was population-based, and data were extracted from a prospective database.

### 4.5 Limitations

The database used was not specifically designed for this study, and utilizing a frailty score instead of the preinjury ASA score would probably better reflect the prognosis. The categorization of rehabilitation into specialized inpatient rehabilitation and no rehabilitation is somewhat unvarnished since some of the patients who did not receive inpatient specialized rehabilitation received some form of rehabilitation at other institutions. Neither the number of patients who received rehabilitation elsewhere nor the type or quality of rehabilitation was included in our registry.

## 5 Conclusion

In Southeast Norway, 96% of patients with cSCI are transferred to the NTC for acute treatment, indicating a need to prioritize referral to the NTC for these patients. Surgery was performed at equal rates regardless of age. Advanced age, especially among octogenarians, was significantly linked to a lack of specialized rehabilitation. Qualified physicians should assess all patients with cSCI for their need of rehabilitation and their potential to benefit from it. If the number of patients who are likely to respond to rehabilitation outnumbers the capacity of the rehabilitation center, we have two choices. Either guidelines for prioritization of patients for rehabilitation should be developed, or the capacity of the rehabilitation centers should be increased.

## Data Availability

The raw data supporting the conclusions of this article will be made available by the authors, without undue reservation.

## References

[1] Barbiellini Amidei C Salmaso L Bellio S Saia M. Epidemiology of traumatic spinal cord injury: a large population-based study. Spinal Cord. (2022) 60:812–9. doi: 10.1038/s41393-022-00795-w, PMID: 35396455 PMC8990493

[2] Bjornshave Noe B Mikkelsen EM Hansen RM Thygesen M Hagen EM. Incidence of traumatic spinal cord injury in Denmark, 1990-2012: a hospital-based study. Spinal Cord. (2015) 53:436–40. doi: 10.1038/sc.2014.181, PMID: 25366529

[3] Johansson E Luoto TM Vainionpaa A Kauppila AM Kallinen M Vaarala E. Epidemiology of traumatic spinal cord injury in Finland. Spinal Cord. (2021) 59:761–8. doi: 10.1038/s41393-020-00575-4, PMID: 33149235 PMC7610166

[4] Montoto-Marques A Trillo-Dono N Ferreiro-Velasco ME Salvador-de la Barrera S Rodriguez-Sotillo A Mourelo-Farina M. Risks factors of mechanical ventilation in acute traumatic cervical spinal cord injured patients. Spinal Cord. (2018) 56:206–11. doi: 10.1038/s41393-017-0005-7, PMID: 29057991

[5] Rau Y Schulz AP Thietje R Matrisch L Frese J Hirschfeld S. Incidence of spinal cord injuries in Germany. Eur Spine J. (2023) 32:601–7. doi: 10.1007/s00586-022-07451-0, PMID: 36371751 PMC9660155

[6] Abedi A Biering-Sorensen F Chhabra HS D’Andrea Greve JM Khan NM Koskinen E. An international survey of the structure and process of care for traumatic spinal cord injury in acute and rehabilitation facilities: lessons learned from a pilot study. BMC Health Serv Res. (2022) 22:1565. doi: 10.1186/s12913-022-08847-w, PMID: 36544168 PMC9768992

[7] Noonan VK Chan E Santos A Soril L Lewis R Singh A. Traumatic spinal cord injury Care in Canada: a survey of Canadian centers. J Neurotrauma. (2017) 34:2848–55. doi: 10.1089/neu.2016.4928, PMID: 28367684 PMC5653141

[8] Fehlings MG Hachem LD Tetreault LA Skelly AC Dettori JR Brodt ED. Timing of decompressive surgery in patients with acute spinal cord injury: systematic review update. Global Spine J. (2024) 14:38S–57S. doi: 10.1177/21925682231197404 38526929 PMC10964893

[9] Chikuda H Koyama Y Matsubayashi Y Ogata T Ohtsu H Sugita S. Effect of early vs delayed surgical treatment on motor recovery in incomplete cervical spinal cord injury with preexisting cervical stenosis: a randomized clinical trial. JAMA Netw Open. (2021) 4:e2133604. doi: 10.1001/jamanetworkopen.2021.33604, PMID: 34751757 PMC8579238

[10] Scivoletto G Morganti B Ditunno P Ditunno JF Molinari M. Effects on age on spinal cord lesion patients’ rehabilitation. Spinal Cord. (2003) 41:457–64. doi: 10.1038/sj.sc.3101489, PMID: 12883544

[11] Scivoletto G Morganti B Molinari M. Early versus delayed inpatient spinal cord injury rehabilitation: an Italian study. Arch Phys Med Rehabil. (2005) 86:512–6. doi: 10.1016/j.apmr.2004.05.021, PMID: 15759237

[12] Putzke JD Barrett JJ Richards JS DeVivo MJ. Age and spinal cord injury: an emphasis on outcomes among the elderly. J Spinal Cord Med. (2003) 26:37–44. doi: 10.1080/10790268.2003.11753659, PMID: 12830968

[13] Furlan JC Hitzig SL Craven BC. The influence of age on functional recovery of adults with spinal cord injury or disease after inpatient rehabilitative care: a pilot study. Aging Clin Exp Res. (2013) 25:463–71. doi: 10.1007/s40520-013-0066-1 23784728

[14] Roquilly A Vigue B Boutonnet M Bouzat P Buffenoir K Cesareo E. French recommendations for the management of patients with spinal cord injury or at risk of spinal cord injury. Anaesth Crit Care Pain Med. (2020) 39:279–89. doi: 10.1016/j.accpm.2020.02.003 32229270

[15] Maharaj MM Stanford RE Lee BB Mobbs RJ Marial O Schiller M. The effects of early or direct admission to a specialised spinal injury unit on outcomes after acute traumatic spinal cord injury. Spinal Cord. (2017) 55:518–24. doi: 10.1038/sc.2016.117 27481092

[16] Rinkaewkan P Kuptniratsaikul V. The effectiveness of inpatients rehabilitation for spinal cord patients in Siriraj hospital. Spinal Cord. (2015) 53:591–7. doi: 10.1038/sc.2015.8, PMID: 25687514

[17] Cao Y Nie J Sisto SA Niewczyk P Noyes K. Assessment of differences in inpatient rehabilitation Services for Length of stay and health outcomes between US Medicare advantage and traditional Medicare beneficiaries. JAMA Netw Open. (2020) 3:e201204. doi: 10.1001/jamanetworkopen.2020.1204, PMID: 32186746 PMC7081121

[18] Malekzadeh H Golpayegani M Ghodsi Z Sadeghi-Naini M Asgardoon M Baigi V. Direct cost of illness for spinal cord injury: a systematic review. Global Spine J. (2022) 12:1267–81. doi: 10.1177/21925682211031190, PMID: 34289308 PMC9210246

[19] Parent S Barchi S LeBreton M Casha S Fehlings MG. The impact of specialized centers of care for spinal cord injury on length of stay, complications, and mortality: a systematic review of the literature. J Neurotrauma. (2011) 28:1363–70. doi: 10.1089/neu.2009.1151, PMID: 21410318 PMC3143414

[20] Andelic N Bautz-Holter E Ronning P Olafsen K Sigurdardottir S Schanke AK. Does an early onset and continuous chain of rehabilitation improve the long-term functional outcome of patients with severe traumatic brain injury? J Neurotrauma. (2012) 29:66–74. doi: 10.1089/neu.2011.1811, PMID: 21864138

[21] Singh A Tetreault L Kalsi-Ryan S Nouri A Fehlings MG. Global prevalence and incidence of traumatic spinal cord injury. Clin Epidemiol. (2014) 6:309–31. doi: 10.2147/CLEP.S68889, PMID: 25278785 PMC4179833

[22] van den Berg ME Castellote JM Mahillo-Fernandez I de Pedro-Cuesta J. Incidence of spinal cord injury worldwide: a systematic review. Neuroepidemiology. (2010) 34:184–92. doi: 10.1159/000279335 20130419

[23] Ding W Hu S Wang P Kang H Peng R Dong Y. Spinal cord injury: the global incidence, prevalence, and disability from the global burden of disease study 2019. Spine (Phila Pa 1976). (2022) 47:1532–40. doi: 10.1097/BRS.0000000000004417, PMID: 35857624 PMC9554757

[24] Bellucci CH Castro Filho JE Gomes CM Bessa Junior J Battistella LR Souza DR. Contemporary trends in the epidemiology of traumatic spinal cord injury: changes in age and etiology. Neuroepidemiology. (2015) 44:85–90. doi: 10.1159/000371519, PMID: 25765118

[25] Hagen EM Eide GE Rekand T Gilhus NE Gronning M. A 50-year follow-up of the incidence of traumatic spinal cord injuries in Western Norway. Spinal Cord. (2010) 48:313–8. doi: 10.1038/sc.2009.133, PMID: 19823192

[26] Vi blir stadig eldre: Statistisk Sentralbyrå; (2020). Available at: https://www.ssb.no/befolkning/artikler-og-publikasjoner/vi-blir-stadig-eldre.

[27] Ryken TC Hurlbert RJ Hadley MN Aarabi B Dhall SS Gelb DE. The acute cardiopulmonary management of patients with cervical spinal cord injuries. Neurosurgery. (2013) 72:84–92. doi: 10.1227/NEU.0b013e318276ee16, PMID: 23417181

[28] Jia X Kowalski RG Sciubba DM Geocadin RG. Critical care of traumatic spinal cord injury. J Intensive Care Med. (2013) 28:12–23. doi: 10.1177/0885066611403270 21482574

[29] Hadley MN Walters BC Grabb PA Oyesiku NM Przybylski GJ Resnick DK. Blood pressure management after acute spinal cord injury. Neurosurgery. (2002) 50:S58–62. doi: 10.1097/00006123-200203001-00012, PMID: 12431288

[30] Merli GJ Crabbe S Paluzzi RG Fritz D. Etiology, incidence, and prevention of deep vein thrombosis in acute spinal cord injury. Arch Phys Med Rehabil. (1993) 74:1199–205. doi: 10.1016/S0003-9993(23)00015-1 8239962

[31] Bellucci CH Wollner J Gregorini F Birnbock D Kozomara M Mehnert U. Acute spinal cord injury—do ambulatory patients need urodynamic investigations? J Urol. (2013) 189:1369–73. doi: 10.1016/j.juro.2012.10.013, PMID: 23069382

[32] Utheim NC Helseth E Stroem M Rydning P Mejlaender-Evjensvold M Glott T. Epidemiology of traumatic cervical spinal fractures in a general Norwegian population. Inj Epidemiol. (2022) 9:10. doi: 10.1186/s40621-022-00374-w, PMID: 35321752 PMC8943974

[33] Kirshblum SC Waring W Biering-Sorensen F Burns SP Johansen M Schmidt-Read M. Reference for the 2011 revision of the international standards for neurological classification of spinal cord injury. J Spinal Cord Med. (2011) 34:547–54. doi: 10.1179/107902611X13186000420242, PMID: 22330109 PMC3232637

[34] Avila MJ Hurlbert RJ. Central cord syndrome redefined. Neurosurg Clin N Am. (2021) 32:353–63. doi: 10.1016/j.nec.2021.03.007, PMID: 34053723

[35] Fassett DR Harrop JS Maltenfort M Jeyamohan SB Ratliff JD Anderson DG. Mortality rates in geriatric patients with spinal cord injuries. J Neurosurg Spine. (2007) 7:277–81. doi: 10.3171/SPI-07/09/277 17877260

[36] Barbara-Bataller E Mendez-Suarez JL Aleman-Sanchez C Sanchez-Enriquez J Sosa-Henriquez M. Change in the profile of traumatic spinal cord injury over 15 years in Spain. Scand J Trauma Resusc Emerg Med. (2018) 26:27. doi: 10.1186/s13049-018-0491-4, PMID: 29622032 PMC5887209

[37] Kannus P Palvanen M Niemi S Parkkari J. Alarming rise in the number and incidence of fall-induced cervical spine injuries among older adults. J Gerontol A Biol Sci Med Sci. (2007) 62:180–3. doi: 10.1093/gerona/62.2.180 17339643

[38] Wang TY Park C Zhang H Rahimpour S Murphy KR Goodwin CR. Management of Acute Traumatic Spinal Cord Injury: a review of the literature. Front Surg. (2021) 8:698736. doi: 10.3389/fsurg.2021.698736, PMID: 34966774 PMC8710452

[39] Hawryluk G Whetstone W Saigal R Ferguson A Talbott J Bresnahan J. Mean arterial blood pressure correlates with neurological recovery after human spinal cord injury: analysis of high frequency physiologic data. J Neurotrauma. (2015) 32:1958–67. doi: 10.1089/neu.2014.3778, PMID: 25669633 PMC4677564

[40] Kwon BK Tetreault LA Martin AR Arnold PM Marco RAW Newcombe VFJ. A clinical practice guideline for the Management of Patients with Acute Spinal Cord Injury: recommendations on hemodynamic management. Global. Spine J. (2024) 14:187S–211S. doi: 10.1177/21925682231202348 PMC10964888 38526923

[41] Berlly M Shem K. Respiratory management during the first five days after spinal cord injury. J Spinal Cord Med. (2007) 30:309–18. doi: 10.1080/10790268.2007.11753946, PMID: 17853652 PMC2031940

[42] Burns AS Marino RJ Kalsi-Ryan S Middleton JW Tetreault LA Dettori JR. Type and timing of rehabilitation following acute and subacute spinal cord injury: a systematic review. Global Spine J. (2017) 7:175S–94S. doi: 10.1177/2192568217703084, PMID: 29164023 PMC5684843

[43] Mahabaleshwarkar R Khanna R. National hospitalization burden associated with spinal cord injuries in the United States. Spinal Cord. (2014) 52:139–44. doi: 10.1038/sc.2013.144, PMID: 24276419

[44] Dicpinigaitis AJ Al-Mufti F Bempong PO Kazim SF Cooper JB Dominguez JF. Prognostic significance of baseline frailty status in traumatic spinal cord injury. Neurosurgery. (2022) 91:575–82. doi: 10.1227/neu.0000000000002088 35944118

[45] Mac-Thiong JM Feldman DE Thompson C Bourassa-Moreau E Parent S. Does timing of surgery affect hospitalization costs and length of stay for acute care following a traumatic spinal cord injury? J Neurotrauma. (2012) 29:2816–22. doi: 10.1089/neu.2012.2503, PMID: 22920942

[46] Aarhus M Mirzamohammadi J Ronning PA Strom M Glott T Rizvi SAM. Time from injury to acute surgery for patients with traumatic cervical spinal cord injury in south-East Norway. Front Neurol. (2024) 15:1420530. doi: 10.3389/fneur.2024.1420530, PMID: 38978812 PMC11228170

[47] Kopp MA Lubstorf T Blex C Schwab JM Grittner U Auhuber T. Association of age with the timing of acute spine surgery-effects on neurological outcome after traumatic spinal cord injury. Eur Spine J. (2022) 31:56–69. doi: 10.1007/s00586-021-06982-2, PMID: 34533643

[48] Chamberlain JD Ronca E Brinkhof MW. Estimating the incidence of traumatic spinal cord injuries in Switzerland: use of administrative data to identify potential coverage error in a cohort study. Swiss Med Wkly. (2017) 147. doi: 10.4414/smw.2017.14430 28488262

[49] Lim V Richard-Denis A Dionne A Maurais G Bourassa-Moreau E Mac-Thiong JM. Does older age affect the likelihood to achieve Normal quality of life after traumatic spinal cord injury? A prospective observational cohort study. J Neurotrauma. (2023) 40:876–82. doi: 10.1089/neu.2022.0025, PMID: 36173098

[50] Sadeghi-Naini M Yousefifard M Ghodsi Z Azarhomayoun A Kermanian F Golpayegani M. In-hospital mortality rate in subaxial cervical spinal cord injury patients: a systematic review and meta-analysis. Acta Neurochir. (2023) 165:2675–88. doi: 10.1007/s00701-023-05720-5, PMID: 37480505

[51] Casper DS Zmistowski B Schroeder GD McKenzie JC Mangan J Vatson J. Preinjury patient characteristics and Postinjury neurological status are associated with mortality following spinal cord injury. Spine (Phila Pa 1976). (2018) 43:895–9. doi: 10.1097/BRS.0000000000002533, PMID: 29280931

[52] Carlile CR Rees AB Schultz JD Steinle AM Nian H Smith MD. Predicting mortality in elderly spine trauma patients. Spine (Phila Pa 1976). (2022) 47:977–85. doi: 10.1097/BRS.0000000000004362 35472062

[53] Brown R DiMarco AF Hoit JD Garshick E. Respiratory dysfunction and management in spinal cord injury. Respir Care. (2006) 51:853–68; discussion 69-70. PMID: 16867197 PMC2495152

[54] Inglis T Banaszek D Rivers CS Kurban D Evaniew N Fallah N. In-hospital mortality for the elderly with acute traumatic spinal cord injury. J Neurotrauma. (2020) 37:2332–42. doi: 10.1089/neu.2019.6912, PMID: 32635809 PMC7585611

